# Antiparasitic potential of *Nephrolepis biserrata* methanol extract against the parasitic leech *Zeylanicobdella arugamensis* (Hirudinea) and LC-QTOF analysis

**DOI:** 10.1038/s41598-020-79094-4

**Published:** 2020-12-16

**Authors:** Muhammad Dawood Shah, Balu Alagar Venmathi Maran, Fatin Khairah Haron, Julian Ransangan, Fui Fui Ching, Sitti Raehanah Muhamad Shaleh, Rossita Shapawi, Yoong Soon Yong, Susumu Ohtsuka

**Affiliations:** 1 Borneo Marine Research Institute, Universiti Malaysia Sabah, Jalan UMS, 88400 Kota Kinabalu, Sabah Malaysia; 2 Xiamen University Malaysia, Jalan Sunsuria, Bandar Sunsuria, 43900 Sepang, Selangor Malaysia; 3 Fisheries Science Laboratory, Setouchi Field Science Center, Graduate School of Integrated Sciences for Life, Hiroshima University, Higashi Hiroshima, Hiroshima Japan

**Keywords:** Biological techniques, Biotechnology

## Abstract

Marine leech *Zeylanicobdella arugamensis* (Piscicolidae), an economically important parasite is infesting predominantly cultured groupers, hybrid groupers and other fish in Southeast Asian countries. In this study, we tested the anti-parasitic potential of a medicinal plant *Nephrolepis biserrata* found in Sabah, East Malaysia against *Z. arugamensis*. Various concentrations of methanol extracts of the plant were tested experimentally against *Z. arugamensis* and disinfestation of the leech from its primary host hybrid groupers. The composition of methanol extract of *N. biserrata* was determined through LC-QTOF analysis. The significant anti-parasitic activity of 100% mortality of leeches was observed with the exposure of *N. biserrata* extracts. The average time to kill the leeches at concentrations of 25, 50 and 100 mg/ml was 25.11 ± 3.26, 11.91 ± 0.99, and 4.88 ± 0.50 min., respectively. Further, at various low concentrations of *N*. *biserrata* 2.5, 5 and 10 mg/ml, hybrid groupers were disinfested in an average time of 108.33 ± 12.65, 65.83 ± 9.70 and 29.16 ± 5.85 min., respectively. The tandem mass spectrometry data from LC-QTOF indicated some hits on useful bioactive compounds such as terpenoids (ivalin, isovelleral, brassinolide, and eschscholtzxanthin), flavonoids (alnustin, kaempferol 7,4′-dimethyl ether, and pachypodol), phenolics (piscidic acid, chlorogenic acid, and ankorine), and aromatic (3-hydroxycoumarin). Thus *N. biserrata* can act as a potential biocontrol agent.

## Introduction

Groupers of the family Serranidae, are acknowledged as economically-valued marine fish in Asian countries^1^. Around 21 species of groupers are cultured in Asia, especially in China, Japan, Thailand, Indonesia, Philippines, and Malaysia for domestic consumption and overseas export^2^. Tiger grouper (*Epinephelus fuscoguttatus*) and giant grouper (*Epinephelus lanceolatus*) are having a high commercial value in Malaysia, the price of these groupers is between USD 12 and 90 per kilogram^3^. To speed up the growth of groupers in aquaculture industry hybrid groupers were started to produce and it demonstrated better growth rate under grow out conditions^3,4^. They utilized feed more efficiently, implies a reduction in the feed cost for commercial farming^4,5^.

A wide variety of parasitic organisms such as protozoans, monogeneans, didymozoid digeneans, nematodes, copepods, and leeches have been reported as causing significant harms to grouper aquaculture^2,6,7^. Leeches are ectoparasites with striated bodies, muscular body wall with two suckers used for feeding and movement. *Zeylanicobdella arugamensis* (Annelida: Hirudinea: Piscicolidae), a marine parasitic leech, distributed throughout the Indian Ocean due to global ecological changes^8^. It is considered as a harmful parasite and affects a large number of fish species, can represent stressors for fish and have been associated with reduced food intake, anti-predator behaviour and decreased growth^9^. They attach in great numbers on the fins, jaws, inside the mouth and eyes of the host fishes^9^. In addition to this, the infestation of ectoparasites also results in the secondary infection of the host by the pathogenic bacteria such as *Vibrio alginolyticus* which further leads to the mortality of the host in a very short period^7,9,10^. Thus, *Z. arugamensis* is a vital threat to the grouper and other species in the aquaculture industry.

Toxic chemicals have been used for the control, especially formalin which are harmful to both fish and human beings^11–14^. In addition, some other chemicals such as copper sulfate, potassium permanganate, and hydrogen peroxide, have also been used, they could affect the water quality and other parameters including health of human beings through accumulation of toxic contents in fish. Hence, researchers and farmers are looking for alternative approaches including the medicinal plants for the eco-friendly treatments^13^. The natural products such as medicinal plants are possessing anti-inflammatory and antipathogenic properties with minimal toxicity level to fish. To minimize the consumption of toxic chemicals plant derived extracts can be used as a natural control agent against parasitic infestation due to the presence of antiparasitic phytochemical compounds such as alkaloids, glycosides, terpenoids etc^15–17^. Hence, research on the development of safe and natural agent for controlling the infestation rate of this species is critical.

*Nephrolepis biserrata* is a perennial fern, belongs to the family Nephrolepidaceae^18^, locally known as “Paku pedang” ^19^. In addition to Malaysia, the plant is also distributed in other South-east Asian countries^20,21^. In this medicinal plant, phytochemical profiling via gas chromatography mass spectrometry (GCMS) and the hepatoprotective properties have been studied^22,23^. Besides, the plant possesses antibacterial and antifungal properties^24^. However, no data have so far been reported concerning the anti-parasitic properties of *N. biserrata* against *Z. arugamensis*. The main objectives of the present study were to evaluate the anti-parasitic activity of *N. biserrata* methanol extract against the leech *Z. arugamensis,* disinfestation of the hybrid groupers (*Epinephelus fuscoguttatus* x *E.lanceolatus*) and chemical composition by LC-QTOF.

## Results

### Anti-parasitic properties of *N. biserrata*

The mortality time of *Z. arugamensis* in formalin and extract-treated groups is indicated in Table 1. The plant treated groups showed the antiparasitic effect in a dose-dependent manner. The time taken for the mortality of the parasitic leeches was lesser in 100 mg/ml dose than 50 and 25 mg/ml of the methanol extract. No mortality was noticed in the normal control group throughout the 720 min observation.

**Table 1:** Mortality time of the leeches at different concentrations of methanol extract of *N. biserrata.*

No.	Group	Mortality time (min) Mean ± S. D	Mortality (%)
1	Normal control	720.00 ± 00	0
2	Positive control (formalin 0.25%) (v/v)	3.68 ± 0.46^a^	100
3	*N. biserrata* (25 mg/ml)	25.11 ± 3.26^a,b^	100
4	*N. biserrata* (50 mg/ml)	11.91 ± 0.99^a,b,c^	100
5	*N. biserrata* (100 mg/ml)	4.88 ± 0.50^a,c,d^	100

Each value represents the mean ± S.D of 6 parasitic leeches per group.

^a^Significance at *p* < 0.05 compared with the normal control group.

^b^Significance at *p* < 0.05 compared with the formalin treated group (0.25% v/v).

^c^Significance at *p* < 0.05 compared with *N. biserrata* (25 mg/ml).

^d^Significance at *p* < 0.05 compared with *N. biserrata* (50 mg/ml).

### Fish disinfestation

The disinfestation time of fishes treated with formalin and methanol extract of *N. biserrata* is shown in Table 2*.* The normal control group is free of leeches. The negative control group indicated no disinfestation of the leeches until 720 min. The plant treated groups indicated the effect in a dose-dependent manner. The time taken for 100% disinfestation of the fishes at a dose of 10 mg/ml is less than 5 and 2.5 mg/ml of the methanol extracts.

**Table 2:** Fish disinfestation time of leeches at different concentrations of methanol extract of *N. biserrata.*

No.	Group	Disinfestation time (min) Mean ± S.D	Disinfestation (%)
1	Normal control	–	–
2	Negative control	720.00 ± 0.00	0
3	Positive control (formalin 0.1%) (v/v)	26.33 ± 1.52^a^	100
4	*N. biserrata* (2.5 mg/ml)	108.33 ± 12.65^a,b^	100
5	*N. biserrata* (5 mg/ml)	65.83 ± 9.70^a,b,c^	100
6	*N. biserrata* (10 mg/ml)	29.16 ± 5.85^a,c,d^	100

Each value represents mean ± S.D of 6 fishes per group.

^a^Significance at *p* < 0.05 compared with the negative control group.

^b^Significance at *p* < 0.05 compared with the formalin treated group (0.1%) (v/v).

^c^Significance at *p* < 0.05 compared with *N. biserrata* (2.5 mg/ml).

^d^Significance at *p* < 0.05 compared with *N. biserrata* (5.0 mg/ml).

### Behaviour of *Z. arugamensis*

**Figure 1:**
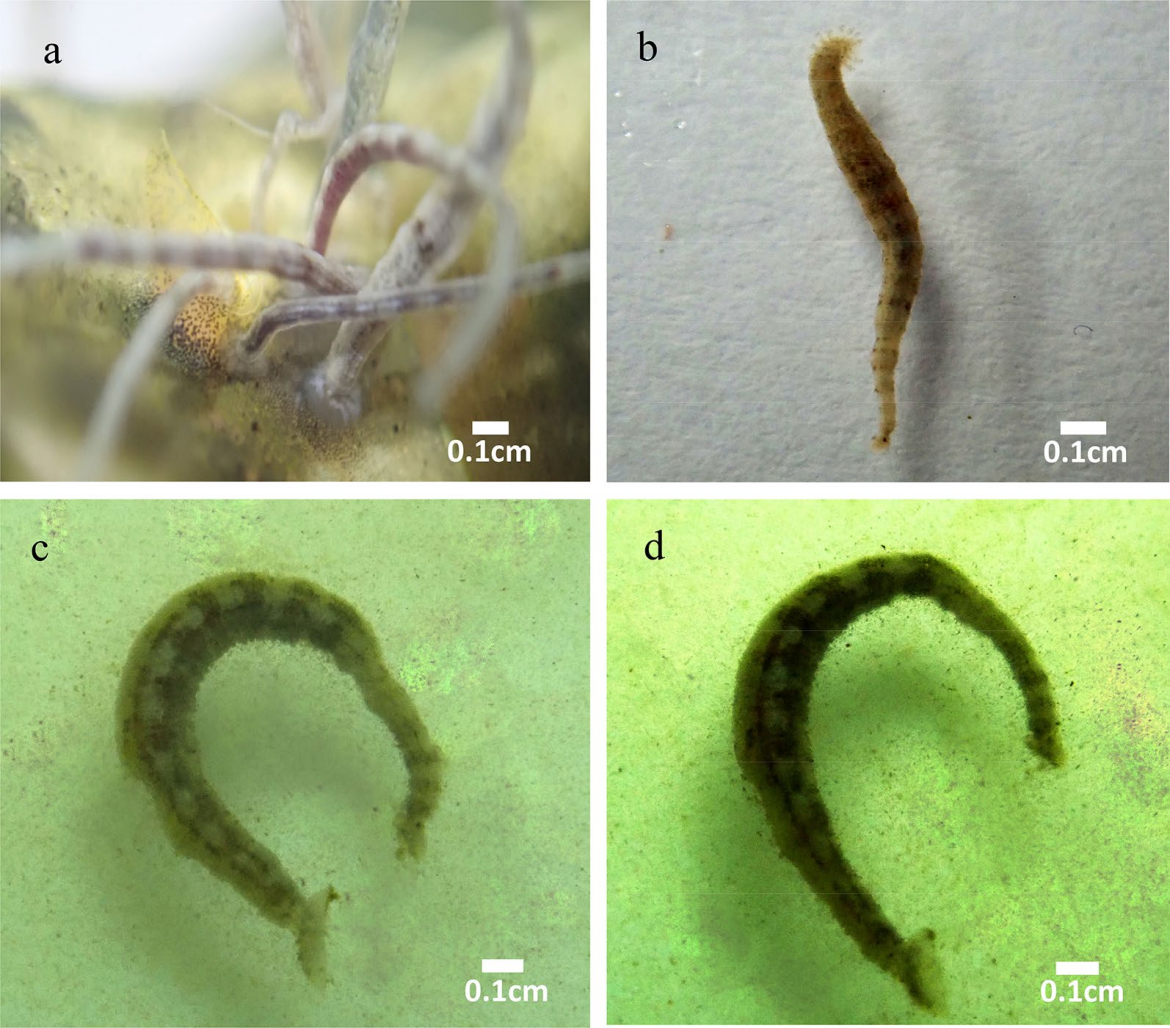
*Zeylanicobdella arugamensis*, (**a**) normal control, (**b**) formalin administered group, (**c**) and (**d**) *Nephrolepis biserrata* methanol extract administered group.

The leeches used their anterior and posterior suckers for locomotion and showed well-organized movement. In the normal control group, the leeches were attached firmly with suckers (Fig. 1a). In the case of formalin, the leeches showed strong swimming for 60 s and it declined sluggishly, after 3 min no movements were noticed. In the formalin treated group, some of the parasitic leeches were able to attach the anterior or posterior sucker to the bottom of the Petri dish before they were dead as compared to the extract-treated group (Fig. 1b). When the leeches were exposed to various concentrations of methanol extract of *N. biserrata*, the movement was not organized and the leeches were not able to use either posterior or anterior suckers for the movement. At 100 mg/ml, the leeches showed vigorous swimming in a zig-zag pattern from 30 to 60 s; then, the swimming gradually reduced; while after 3–4 min, the movement stopped, and the leeches were paralyzed and dead, indicated no movement after physical touch. At 50 mg/ml, fast-swimming was noticed for 30 s and it decreased slowly. After 7 min, no movements were noticed, and the leeches moved only with a physical touch before they were dead. At 25 mg/ml, strong movements were noticed for 30 s and it lessened gradually, after 18 to 25 min the movements were stopped (Fig. 1c, d).

### Behaviour of hybrid groupers

**Figure 2:**
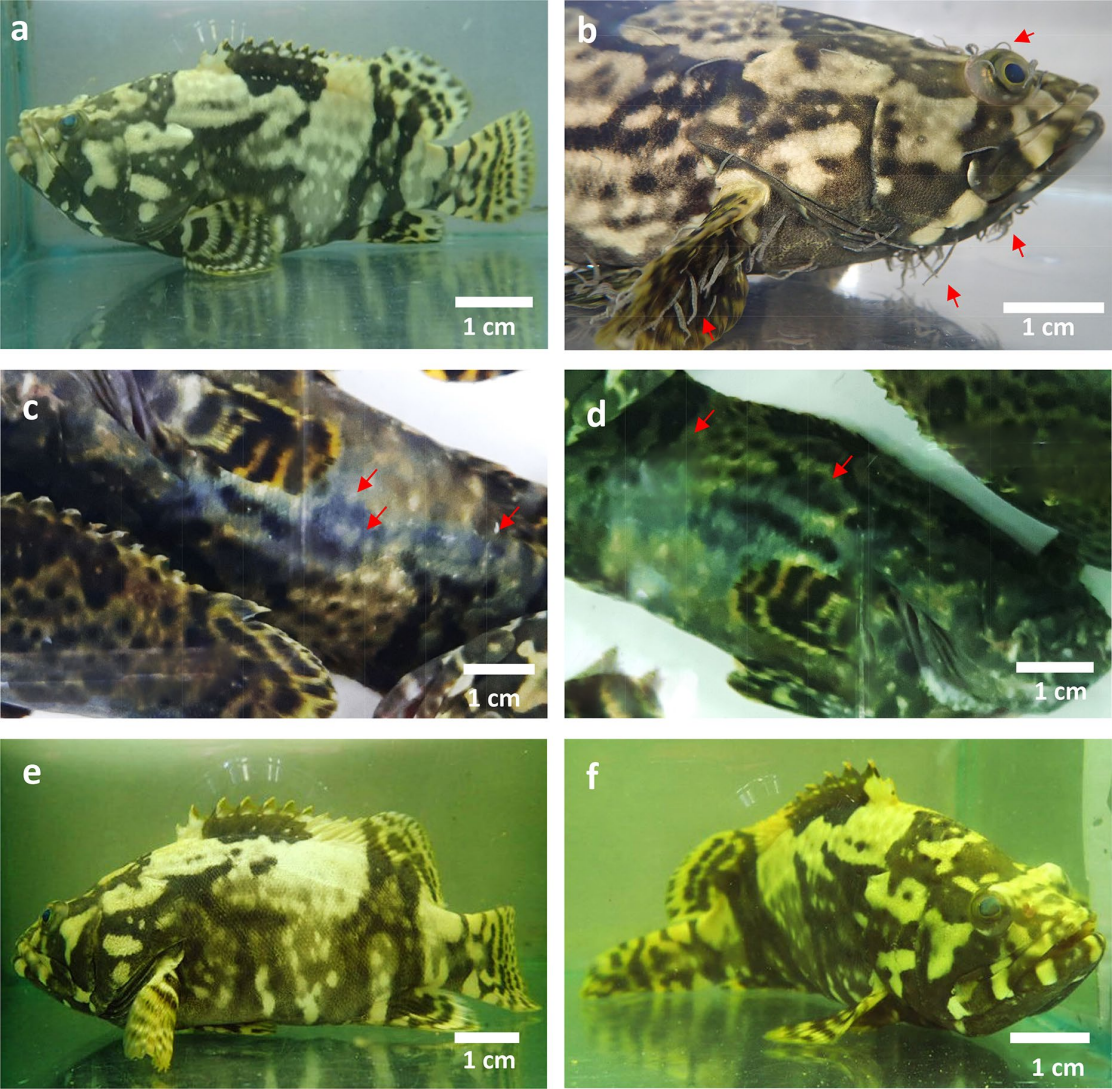
Hybrid groupers (*Epinephelus fuscoguttatus* x *E.lanceolatus*) (**a**) normal control, (**b**)  positive control, highly infested with parasitic leeches, the arrow indicates the prevalence of the leeches on the head, eyes and fins area, (**c,d**) formalin (0.1%) treated group, arrows indicate the appearance of wounds on the body surface due to the aggressive swimming and stress induced by the chemical, (**e,f**)  *Nephrolepis biserrata* methanol extract administered group, no wounds or leeches were noticed on the surface of the fishes after the treatment.

Fishes in the normal control group showed normal swimming activity (Fig. 2a) and in the negative control group they were weak (Fig. 2b). Fishes treated with 0.1% formalin showed extreme aggressive behaviour throughout the treatment. Around body areas of the formalin treated groups were found with wounds (Fig. 2c, d) as compared to the plant treated groups. However, active swimming was noticed in the group exposed to 10 mg/ml of the plant extract for the first 1 to 5 min later, they were relaxed. The fishes exposed to 5 mg/ml of the extract showed active swimming for 1 to 2 min while no active swimming was noticed in the fishes treated with 2.5 mg/ml of the plant extract. In the end, all the fishes treated with plant extracts were relaxed (Fig. 2e, f).

### Physicochemical parameters of leeches treated solutions

The water quality parameters of the control groups and methanol extract groups solutions applied for the anti-parasitic assays are indicated in Table 3. All parameters determined were remained constant except with a change in pH value of the extract solution as compared to the control solutions. The pH ranged from 4.4 to 4.15 in the methanol solutions of *N. biserrata.* The change in pH of the plant solution could be due to the presence of different bioactive compounds with acidic nature.

**Table 3:** Water quality parameters of the solutions for the treatment of leeches.

No.	Water parameters		Concentrations			
	Groups	Normal control	Positive control (formalin 0.25%) (v/v)	*N. biserrata* (mg/ml)		
				(25)	(50)	(100)
1	Temperature (°C)	24.76	26.16	24.9	24.9	25.1
2	pH	7.89	7.24	4.31	4.20	4.15
3	Salinity (ppt)	30.9	30.9	30.9	30.9	30.9
4	Dissolved oxygen (mg/l)	5.50	5.00	5.28	5.40	5.50

### Physicochemical parameters of hybrid groupers treated solutions

The water quality parameters of the normal, negative and positive control group solutions and methanol extract solutions applied for the fish disinfestation are indicated in Table 4. All parameters determined remained constant. The pH of the methanol extract solutions was maintained above 7 with the addition of sodium bicarbonate (I mg/ml) to provide an alkaline environment to the fishes.

**Table 4:** Water quality parameters of the solutions applied for the fish treatment.

No.	Water parameters			Concentrations			
	Groups	Normal control	Negative control	Positive control (formalin 0.1%) (v/v)	*N. biserrata* (mg/ml)		
					(2.5)	(5)	(10)
1	Temperature (°C)	28.0	28.0	28.0	28.0	28.0	28.0
2	pH	7.13	7.36	7.68	7.04	8.04	7.15
3	Salinity (ppt)	30.29	33.34	30.51	33.03	33.03	33.39
4	Dissolved oxygen (mg/l)	5.16	5.56	4.91	5.09	5.63	5.87

### LC-QTOF analysis and metabolites identification

**Figure 3:**
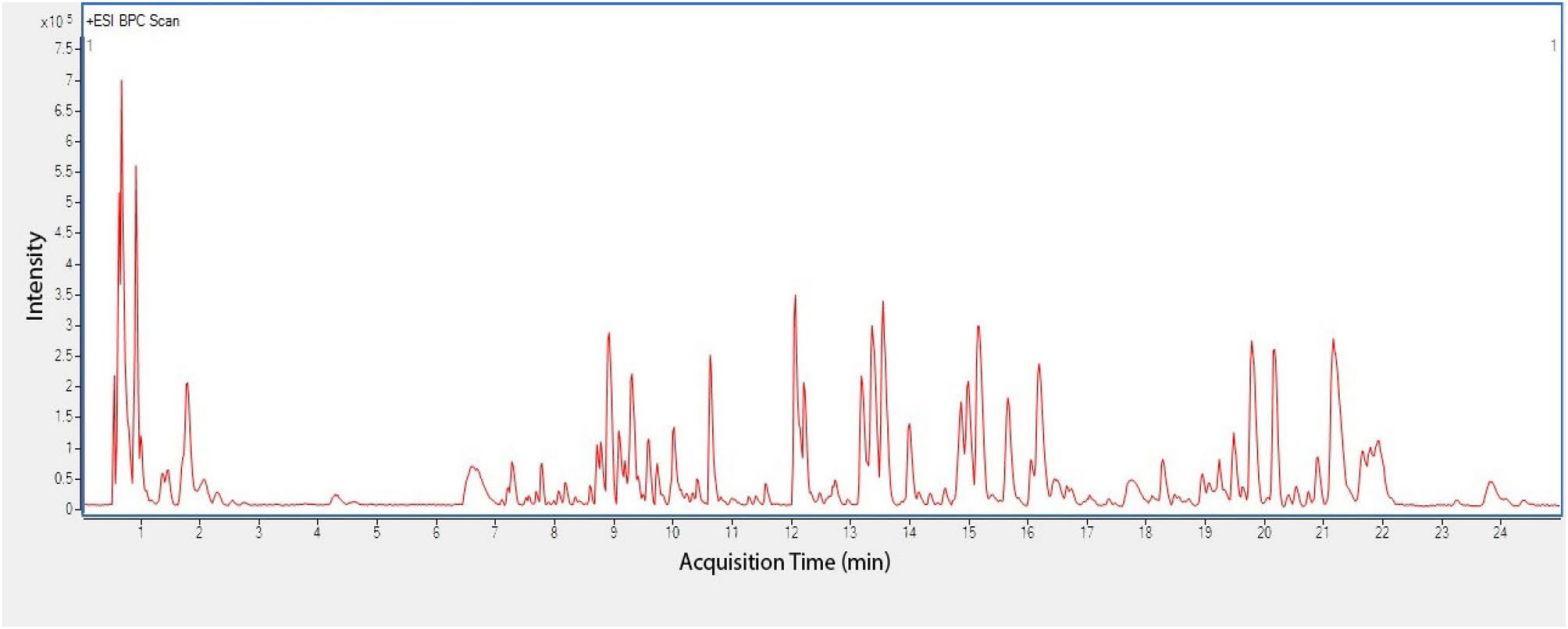
Base peak chromatogram (BPC) of the methanol extract of *Nephrolepis biserrata.*

A total of 77 compound’s features were extracted and determined for their molecular formula by executing Molecular Feature Extraction (MFE) algorithm in Agilent MassHunter software. Among these 77 metabolites, 18 of them were successfully matched with the METLIN databases and previously reported in plant extract (Table 5; Fig. 3).

**Table 5:** Matched metabolites in methanol extract of *N. biserrata.*

Retention time (min)	Mass to charge ratio (*m/z*)	Formula	Mass error (ppm)	Matched metabolites	References
0.766	236.1497	C_10_H_18_O_5_	− 1.91	2-Hydroxydecanedioic acid	^40^
0.869	130.0501	C_5_H_7_NO_3_	− 1.71	Pyroglutamic acid	^41^
0.898	274.0927	C_11_H_12_O_7_	− 2.92	Piscidic Acid	^42^
7.357	355.1027	C_16_H_18_O_9_	− 1.25	Chlorogenic Acid	^34^
8.817	163.0388	C_9_H_6_O_3_	0	3-Hydroxycoumarin	^35^
9.112	197.1169	C_11_H_16_O_3_	1.72	Benzenemethanol, 2-(2-aminopropoxy)-3-methyl	^43^
11.609	329.1029	C_18_H_16_O_6_	− 1.79	Alnustin	^44^
12.767	336.2172	C_19_H_29_NO_4_	− 1.2	Ankorine	^45^
13.378	315.0867	C_17_H_14_O_6_	− 1.26	Kaempferol 7,4′-dimethyl ether	^46^
13.437	345.0975	C_18_H_16_O_7_	− 1.57	Pachypodol	^47^
13.965	249.1489	C_15_H_20_O_3_	− 1.16	Ivalin	^48^
14.856	277.2163	C_18_H_28_O_2_	0.1	9,13-Octadecadiynoic acid	^36^
15.061	277.2164	C_18_H_28_O_2_	− 0.47	9,13-Octadecadiynoic acid	^49^
15.216	233.1539	C_15_H_20_O_2_	− 1.14	Isovelleral	^37^
16.138	295.2266	C_18_H_30_O_3_	0.09	17-Hydroxy-linolenic acid	^50^
16.511	498.3794	C_28_H_48_O_6_	− 0.19	Brassinolide	^51^
19.427	282.2795	C_18_H_35_NO	− 0.88	Oleamide	^52^
22.004	567.4204	C_40_H_54_O_2_	− 1.31	Eschscholtzxanthin	^53^

## Discussion

Ectoparasitic leech (*Z. arugamensis*) provided a significant threat to the grouper aquaculture facilities in Malaysia^8,25^. To control the leech infestation, formalin and other chemicals have been utilized by fish farmers, which cannot provide a conducive environment for the eco-friendly aquaculture policy^14^. On the other hand, plant-based extracts can be used as a safe and natural treatment for the parasite infestations due to the presence of antiparasitic secondary metabolites^16^. In addition to this, the secondary metabolites present in the plant extract also play an important role in the enhancement of growth and immunity of cultured species. The olive oil leaf extract has been reported to boost the activation of intestinal digestive enzyme activity and the expression of genes related with growth in brain, liver, muscle and other vital tissues of common carp *Cyprinus carpio*^26^*.* In the present study, we determined the potential of antiparasitic activity of the local medicinal plant *N. biserrata*. The mortality time of the leeches after the exposure at various concentrations of the plant extracts in a dose-dependent manner has been elucidated (Table 1). Average time of 4.88 ± 0.50 min., was taken to achieve 100% mortality of the leeches in the group treated with 100 mg/ml of the plant extract followed by 11.91 ± 0.99 min. (50 mg/ml) and 25.11 ± 3.26 min. (25 mg/ml). Our results are in comparison with the recently published data regarding the anti-parasitic activity of *Dillenia suffruticosa*^27^. The methanol extract of *D. suffruticosa* showed antiparasitic activity against *Z. arugamensis* with 100% mortality at the concentrations of 100 mg/ml (14.39 ± 3.75 min), 50 mg/ml (32.97 ± 9.29 min) and 25 mg/ml (41.77 ± 5.40 min). In the present study, the anti-parasitic potential of the methanol extract of *N. biserrata* was almost 3 times faster than *D. suffruticosa* methanol extract^28^. In another study, the parasitic leech *Piscicola geometra* from the infested cobia fish exposed for 96 h to various dilutions of the extracts of *Scutellaria baicalensis* and *Morinda citrifolia* and obtained 100% and 80% mortalities, respectively^28^. Further *P. geometra* treated with roots and stem bark extract of *Tetracera alnifolia* plant and indicated a significant effect on the rates of the mortality of the leech after 24 h^29^. The effectiveness of various concentrations of *Nicotiana* spp.*, Zanthoxylum alatum*, and *Solanum khasianum* extracts was tested against *Hirudinaria manillensis* leech*.* All leeches were dead at an average time of 2.11 ± 0.11 min. (*Nicotiana* spp.), 3.00 ± 0.33 min. (*Z. alatum*) and 24.89 ± 2.34 min. (*S. khasianum*)^30^.

Low and suitable concentrations such as 2.5, 5 and 10 mg/ml of the plant extracts were administered for fish disinfestation to avoid the fishes from stress. The disinfestation of fishes was obtained in a dose-dependent manner (Table 2). Groups of infested fishes treated with 10 mg/ml of the methanol extract were disinfested in less than 30 min. Similar results were noticed in the group treated with 5 and 2.5 mg/ml of the extract in the period of 65 and 108 min., respectively. The infested fishes treated with 0.1% formalin took about 26 min. to get rid of the parasitic leeches. According to statistical analysis, no significant differences were noticed in the disinfestation time of the formalin (0.1%) and plant treated group (10 mg/ml). This showed that the treatment effect of the higher concentration of the methanol extract of *N. biserrata* (10 mg/ml) was the same as that of formalin (0.1%) (v/v). Our results are in agreement with the anti-parasitic activity of solvent extract (petroleum ether, chloroform, ethyl acetate, methanol) and aqueous extracts of plants, *Radix angelicae pubescentis*, *Fructus bruceae*, *Caulis spatholobi*, *Semen aesculi*, and *Semen pharbitidis* against the monogenean *Dactylogyrus intermedius* in goldfish^31^. Among the experimental extracts, the methanol and aqueous extracts of *S. aesculi* were noticed to be more effective after 48 h of exposure, followed by methanol extracts of *Fructus bruceae*, *Radix angelicae pubescentis*, *Caulis spatholobi*, and *Semen pharbitidis*^31^.

In the present study, for the comparison of the anti-parasitic effect of the plant extracts, we used formalin as a positive control, since it is vastly utilized for the removal of parasites in aquaculture facilities^14^. Besides, other chemicals such as trichlorfon, hydrogen peroxide and copper sulfate have also been used as an anti-parasitic agent^32^. In Malaysia, other drugs like benzalkonium chloride, acriflavine, malachite green, hypochlorite and poly-vinyl pyrrolidine are also utilized for the removal of parasites in aquaculture facilities^33^. These chemicals are harmful to fish and their consumers^14,33^. However, the methanol extract of *N. biserrata* could be obtained from a natural source with no known side effects^23^.

In the current study, LC-QTOF was employed to profile the methanol extract of *N. biserrata* and detected some interesting metabolites (Table 5). Current tandem mass spectrometry data from LC-QTOF showed some hits on such as terpenoids (ivalin, isovelleral, brassinolide, and eschscholtzxanthin), flavonoids (alnustin, kaempferol 7,4′-dimethyl ether, and pachypodol), phenolics (piscidic acid, chlorogenic acid, and ankorine), and aromatic (3-hydroxycoumarin), etc. Some of these compounds were reported possessing anti-parasitic characteristics, such as chlorogenic acid^34,35^, ivalin^36^ and isovelleral^37^. The chlorogenic acid was reported to possess anti-parasitic activities on vertebrate parasites *Trypanosoma brucei rhodesiense* and *Leishmania donovani* with IC_50_ values of 18.9 µg/ml and 7.0 µg/ml, respectively^34^. Ivalin was also reported with stronger anti-parasitic activity on *Trypanosoma brucei rhodesiense* and *Trypanosoma cruzi* (IC_50_: 1.9 µg/ml and 6.2 µg/ml, respectively)^36^. On the other hand, isovelleral was found significantly inhibited germination of plant-parasitic fungus *Alternaria brassicicola* O-264 isolates conidial at as low as 0.05 ppm of vapour concentration^37^. However, anti-parasitic properties of the detected bioactive compounds in *N. biserrata* have not been reported on marine parasites hence their efficacies are pending for discovery. The strong antiparasitic effect of the methanol extracts of *N. biserrata* could be due to the presence of the above-mentioned metabolites^34–37^.

Our study proved that administration of the methanol extract of *N. biserrata* showed a strong anti-parasitic activity with 100% mortality against the marine leech *Z. arugamensis* parasitic on hybrid groupers in Malaysia. The exposure of the leech-infested hybrid groupers to methanol extracts of *N. biserrata* at a higher concentration (10 mg/ml) also resulted in the removal of parasitic leeches in less than 30 min. The tandem mass spectrometry data from LC-QTOF indicated some hits on terpenoids, flavonoids, phenolics and aromatic. The antiparasitic properties of *N. biserrata* could be due to the presence of various bioactive compounds. Some of the compounds have already been reported with strong anti-parasitic properties. It indicates that *N. biserrata* has a strong potential to act as a biocontrol agent against leech infestation in hybrid groupers. However, further study on the purification and isolation of the pure bioactive compounds responsible for the antiparasitic properties is imperative.

## Materials and methods

### Chemicals

Formalin (37% aqueous formaldehyde solution) and sodium bicarbonates were purchased from Sigma, Leica, Microsystem, and Germany. HPLC grade methanol was obtained from Merck (Darmstadt, Germany). LCMS-grade acetonitrile was purchased from J. T. Baker (Philipsburg, NJ, USA). Deionized water was acquired using Milli-Q system (Merck, Darmstadt, Germany) at resistivity of > 18.2 MΩ cm. Reference mass solution containing 5.0 mM of purine and 2.5 mM of Hexakis [1H,1H,3H-tetrafluoropropoxy] phosphazine, was procured from Agilent Technologies (Santa Clara, CA, USA). LCMS-grade formic acid (HCOOC) was acquired from Fisher Scientific (Fair Lawn, NJ, USA). Polyvinylidene fluoride (PVDF) syringe filters with 0.22 µm pore size and 13 mm diameter were purchased from Merck (Darmstadt, Germany).

### Plant collection

The leaves and stem of the *N. biserrata* were obtained from lowlands of Papar (5.7346° N, 115.9319° E.), Kota Kinabalu, Sabah, Malaysia. The identification of the plant was carried out and a voucher specimen (MDS-001) was deposited at the herbarium of the Institute for Tropical Biology and Conservation, Universiti Malaysia Sabah, Kota Kinabalu, Malaysia.

### Solvent extraction

The leaves and stem of the plant were washed with distilled water and oven-dried in an oven at 37 °C for 3 days. The dried plant was grounded to a fine powder using a mechanical grinder and stored in an airtight container. About 60 g of dry powder of the plant was extracted with HPLC grade methanol (300 ml) using an orbital shaker (25–30 °C) for 3 days. The supernatant was filtered through Whatman No. 41 filter paper by vacuum and the fresh solvent was added to the samples, which were extracted for another 3 days. The methanol residues were removed from the extract using a vacuum rotary evaporator (R-215, BUCHI, Switzerland). The sample was kept at − 80 °C for 24 h and then lyophilized using a freeze drier (Freezon 12, Labconco, USA). The freeze-dried sample (4.80 g) was then stored in the freezer for further studies.

### Anti-parasitic bioassay

Parasitic leeches were collected from the aquaculture facilities of Universiti Malaysia Sabah. The leeches were identified based on their morphological features^10^. Adult leeches were selected, divided into 5 groups (6 leeches per group) and each group was provided with six leeches in a Petri dish.

- Group 1: Normal control, treated with seawater only (Fig. 1a).
- Group 2: Positive control, treated with formalin (0.25% v/v) solution (Fig. 1b).
- Groups 3, 4 and 5: Treated with 25, 50 and 100 mg/ml of the methanol extracts of *N. biserrata,* respectively*.*

During the challenge, mortality time was recorded using a stopwatch for 720 min^27^. The experiment was performed in triplicate.

### Observation on the behaviour of leeches

The changes in the behaviour of leeches were observed visually after exposure to formalin and different concentrations of plant extract as compared to the normal control group.

### Fish disinfestation

Hybrid groupers (*Epinephelus fuscoguttatus* x *E.lanceolatus*) (TL: 15–20 cm) were obtained from the hatchery facilities of University Malaysia Sabah. All animals were treated humanely and well maintained under standard ethical principles as per university regulations (UMS/IP7.5/M3/4–2012). All experimental protocols were approved by the University Malaysia Sabah committee. They were acclimatized for 2 weeks before the start of the experiment. Later, all fishes except normal control group were infested with leeches for one week. Further, they were divided into the following groups (6 fishes per group).

- Group 1: Normal control, treated with seawater only, no leech infestation (Fig. 2a).
- Group 2: Negative control, infested with leeches and no treatment was applied (Fig. 2b).
- Group 3: Positive control, infested + treated with formalin (0.1% v/v) solution.
- Groups 4, 5 and 6: Infested + treated with 2.5, 5.0 and 10 mg/ml of the methanol extract of *N. biserrata,* respectively*.*

During the experiment, the time required for the complete removal of all the leeches (disinfestation time) from the treated fishes was recorded using a stopwatch.

### Observation on the behaviour of hybrid groupers

The changes in the behaviour of the hybrid groupers were monitored after exposure to formalin and different concentrations of the plant extracts in comparison to the normal control group.

### Liquid chromatography quadrupole time-of-flight (LC-QTOF) acquisition

The methanol extract of *N. biserrata* was analyzed using an Agilent 1290 Infinity LC system coupled with an Agilent 6520 QTOF mass spectrometry system. A 2.0 µl sample was injected into a reversed-phase column, namely Agilent Zorbax Eclipse XDB-C18 (narrow bore, 2.1 mm × 150 mm × 3.5 µm; Agilent Technologies, Santa Clara, CA, USA). The column was maintained at 25 °C at a flow rate of 500µL/min during analysis. The mobile phases were composed of solvent A (H_2_O—0.1% HCOOH) and solvent B (acetonitrile—0.1% HCOOH). The gradient elution program was initiated at 5% of solvent B for 5 min, then from 5 to 100% solvent B in 15 min. and kept for 5 min. Later, the column was conditioned as initial for 5 min. prior to next injection.

The mass spectrometry (MS) signals were acquired as described by Ling et al. (2018)^38^ with minor modifications. Briefly, voltage for positive electrospray ionization was changed to 4 kV, while the remaining settings for ion source were maintained. The MS was calibrated with Tuning Mix (Agilent Technologies, Santa Clara, CA, USA) before each batch analysis. Internal mass calibration standards, purine (*m*/*z* 121.0508) and hexakis-(1H, 1H, 3H-tetrafluoropropoxy)-phosphazine (*m*/*z* 922.0097), were introduced throughout the runs for automated mass correction^39^.

### Identification of metabolites

Automated tandem mass spectrometry (auto-MSMS) was employed for metabolites matching, where precursor ions were selected consistently and fragmented throughout the data acquisition. Briefly, two major precursor ions were selected from each MS scan for fragmentation with nitrogen gas at a collision energy of 30 eV. Data acquisition for product ions was set between an *m/z* of 50 and 1500. Acquired data were processed and identified using Agilent MassHunter Qualitative Workflows software (version B.08.00), which is equipped with MassHunter METLIN Metabolite PCD/PCDL (Personal Compound Database and Personal Compound Database and Library).

### Statistical analysis

Data analysis was carried out using IBM SPSS Statistics 25 Window package (IBM, Armonk, NY, US). Significant differences between groups were investigated using one-way analysis of variance (ANOVA) followed by Tukey’s test. All data points were shown as mean ± standard deviation (S.D.). *p* values < 0.05 were viewed as significant^27^.
